# Phospholipase D2 targeted by miR‐5132‐5p alleviates cerulein‐induced acute pancreatitis via the Nrf2/NFκB pathway

**DOI:** 10.1002/iid3.831

**Published:** 2023-05-29

**Authors:** Hailong Wu, Hao Chen, Rui Zhou

**Affiliations:** ^1^ Department of General Surgery Wuhan Fourth Hospital Wuhan Hubei China; ^2^ Department of Neurosurgery Wuhan Fourth Hospital Wuhan Hubei China

**Keywords:** acute pancreatitis, cerulein, miR‐5132‐5p, PLD2

## Abstract

**Background:**

Acute pancreatitis (AP) is an inflammatory process unexpectedly occurring in the pancreas, imposing a substantial burden on healthcare systems. Herein, we aimed to clarify the mechanism of action of phospholipase D2 (PLD2) in cerulein‐treated AR42J cells, affording valuable insights into the treatment of AP.

**Methods:**

The levels of PLD2, miR‐5132‐5p, inflammatory factors (interleukin [IL]−10, IL‐6, and tumor necrosis factor‐α), caspase‐3 activity, and apoptosis‐related proteins (Bax and Bcl‐2) in cerulein‐treated AR42J cells were detected using reverse transcription‐quantitative polymerase chain, caspase‐3 activity, and Western blot analysis. Protein levels of nuclear Factor erythroid 2‐Related Factor 2 (Nrf2) and nuclear factor‐k‐gene binding (NF‐κB) were detected by Western blot analysis. TargetScan predicted upstream microRNAs (miRNAs) of PLD2, and the interaction between miR‐5132‐5p and PLD2 was verified using a luciferase assay.

**Results:**

In cerulein‐treated AR42J cells, PLD2 levels were downregulated, while miR‐5132‐5p expression was upregulated. Overexpression of PLD2 attenuated the cerulein‐mediated facilitatory effect on inflammation and apoptosis in AR42J cells by regulating the Nrf2/NFκB pathway. Luciferase reporter analysis revealed that miR‐5132‐5p targeted PLD2, and miR‐5132‐5p negatively regulated PLD2. Upregulation of miR‐5132‐5p expression exacerbated inflammation and apoptosis and reversed the protective effect of PLD2 overexpression on AP.

**Conclusion:**

PLD2 targeted by miR‐5132‐5p can attenuate cerulein‐induced AP in AR42J cells via the Nrf2/NFκB pathway, providing therapeutic targets for patients with AP.

## Graphical abstract

Phospholipase D2 targeted by miR‐5132‐5p could attenuate cerulein‐induced acute pancreatitis, specifically by weakening the promoting effect of cerulein on inflammation and apoptosis of AR42J cells.

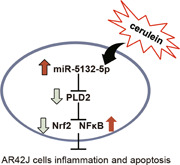

## 1 INTRODUCTION

Acute pancreatitis (AP) is an inflammatory disease that may induce organ failure, secondary pancreatic infection, or peripancreatic necrosis and has been associated with a high mortality rate of up to 20% of patients.^1^ Globally, AP affects millions of individuals, with particularly severe cases causing major clinical and economic burdens in the United States.^2^, ^3^ Despite considerable attempts, the pathogenesis of AP remains poorly understood.^4^ In‐depth study and understanding of the pathogenesis of AP, along with the search for valuable therapeutic strategies, remain of substantial theoretical and clinical significance to treat AP.

Phospholipase D2 (PLD2) belongs to the phospholipase family that catalyzes the degradation of phosphatidylcholine to phosphatidylcholine and choline and participates in several cellular processes, including apoptosis, survival, migration, and adhesion.^5^ PLD2 has been associated with the pathogenesis of various diseases, including vascular, immunological, and neurological diseases.^6^ Blocking PLD2 can improve intestinal mucosal inflammation in inflammatory bowel disease.^7^ In addition, PLD2 plays a crucial role in cell responses to tissue injury, such as acute inflammatory disease.^8^ Moreover, Hwang et al.^9^ have found that PLD2 drives the transport of macrophages out of the vascular system during AP. These data suggest that PLD2 is involved in mediating inflammatory diseases such as AP. However, the role of PDL2 in regulating the pathogenesis of AP remains unclear.

In‐depth exploration of microRNAs (miRNAs) has opened new avenues for AP diagnosis and treatment.^10^ miRNAs are small noncoding RNAs that regulate mRNA expression by inhibiting translation.^11^ Growing evidence suggests that abnormal miRNA expression is involved in inflammatory and invasive diseases, including AP, thereby regulating changes in key physiological functions.^12^, ^13^ For example, miR‐218a‐5p inhibits intestinal cell apoptosis induced by severe AP and improves intestinal dysfunction caused by severe pancreatitis.^14^ miR‐155‐5p was found to be significantly downregulated in AP mice and regulated AP development.^15^ However, the miR‐5132‐5p regulatory mechanism in AP remains elusive.

The objective of the present study was to investigate the role of PLD2 in AP by establishing an in vitro AP model using AR42J cells treated with cerulein. By undertaking functional experiments and targeting analysis, we hypothesized that PLD2 targeted by miR‐5132‐5p could alleviate inflammatory injury and apoptosis in AP. We believe that the findings of the present study will provide novel insights into the diagnosis and treatment of AP.

## 2 MATERIALS AND METHODS

### 2.1 Cell culture and model establishment

Rat pancreatic AR42J cells, purchased from ATCC, were maintained in an incubator under 5% CO_2_ at 37°C and supplemented with F‐12K medium containing 10% fetal bovine serum (Gibco). To establish an in vitro model of pancreatitis, 10 nM cerulein was added to AR42J cells, namely the cerulein (CER) group.^16^ In addition, AR42J cells were cultured with the same amount of phosphate‐buffered saline, deemed the control (CON) group.

### 2.2 Cell transfection

PcDNA‐based PLD2 overexpression plasmid (PLD2‐OE)) and nontarget plasmid negative control (pcDNA) were obtained from GeneChem. MiR‐5132‐5p mimic and disrupted oligonucleotides were obtained from SwitchGear Genomics. In accordance with the manufacturer's instructions, AR42J cells were transfected with the indicated 20 nM oligonucleotide or 10 ng plasmid using Lipofectamine 3000 (Thermo Fisher Scientific). After transfection for 48 h, cell transfection efficiency was evaluated by reverse transcription‐quantitative polymerase chain (RT‐qPCR).

### 2.3 RT‐qPCR analysis

Total RNA was extracted using an RNeasy Mini Kit (Qiagen) in accordance with the manufacturer's instructions. Total RNA was reverse‐transcribed using the PrimeScript First Strand cDNA Synthesis Kit (Takara), and miRNAs were synthesized into cDNA using the miRcute miRNA First Strand cDNA Synthesis Kit (Tiangen). RT‐qPCR was performed on a Roder‐Gene Q Instrument (Qiagen) using SYBR Green PCR Master Mix or the miScript SYBR Green PCR Kit (Qiagen). The expression of labeled mRNA and miR‐5132‐5p was normalized using GAPD or U6 and calculated using the $2^{‐\Delta \Delta C_{t}}$ method. PCR primers used are listed in Table 1.

**Table 1:** The primer sequences are given in Table 1.

Primer name	Sequence (5'−3')
TNF‐α‐FW	ACTGAACTTCGGGGTGATTG
TNF‐α‐RW	GCTTGGTGGTTTGCTACGAC
IL‐6‐FW	TGATGGATGCTTCCAAACTG
IL‐6‐RW	GAGCATTGGAAGTTGGGGTA
IL‐10‐FW	GCTCTTACTGGCTGGAGTGAG
IL‐10‐RW	CTCAGCTCTCGGAGCATGTG
PLD2‐FW	CTATGGGGACCTGAACTC
PLD2‐RW	GACTTTGTGTCTCTGGAGGTC
miR‐203a‐3p‐FW	GCGCGTGAAATGTTTAGGAC
miR‐203a‐3p‐RW	GTGCAGGGTCCGAGGT
miR‐5132‐5p‐FW	GCGTGGGGTGGTGGACT
miR‐5132‐5p‐RW	CTGGAGCGCGCGGGC
β‐actin‐FW	AGAAGAGCTATGAGCTGCCTGACG
β‐actin‐RW	CTTCTGCATCCTGTCAGCGATGC
U6‐FW	CTCGCTTCGGCAGCACA
U6‐RW	AACGCTTCACGAATTTGCGT

### 2.4 Caspase‐3 activity assay

A Caspase‐3 Assay Kit (Cell Signaling Technology) was used to determine caspase‐3 activity. Briefly, AR42J cells were lysed and incubated with 5 µL DEVD‐pNA bound Caspase‐3 substrate for 1 h at 37°C. Caspase‐3 activity was measured at 405 nm using a microplate reader (Bio‐Rad).

### 2.5 Western blot analysis

The protein concentration in AR42J cells was determined using the BCA method after the cells were lysed using RIPA buffer. For detecting nuclear Factor erythroid 2‐Related Factor 2 (Nrf2) and nuclear factor‐k‐gene binding (NFκB) protein levels, a nuclear and cytoplasmic protein extraction kit (Beyotime) was used to isolate proteins from the nucleus and cytoplasm. An equivalent amount of protein (50 µg) was resolved by 12% sodium dodecyl sulfate‐polyacrylamide gel electrophoresis (SDS‐PAGE) and transferred to a polyvinylidene fluoride membrane. The membrane was sealed with 0.1% tween‐20% and 5% bovine serum albumin (Sigma‐Aldrich) for 1 h and left overnight at 4°C with the following antibodies purchased from Abcam: Bax (1:1000; ab32503), Bcl‐2 (1:1000; ab32124), PLD2 (1:500; ab78907), NF‐κB (1:1000; ab32360), Nrf2 (1:1000; ab92946), or β‐actin (1:2000; ab8227). After rinsing with Tris‐buffered saline containing 0.05% Tween, the membrane was incubated with horseradish (HRP)‐conjugated secondary antibody (1:2000; ab6721) at room temperature for 2 h. Immunoreactive bands were detected using an enhanced chemiluminescence detection system (Pierce Biotech), and band intensities were measured and quantified by densitometric analysis using the Gel‐Pro analyzer (version 4.0; Gel Media Cybernetics).

### 2.6 Luciferase reporter assay

By undertaking bioinformatics analysis, the putative miR‐5132‐5p binding site in the PLD2 3'‐UTR was predicted using Targetscan. A human PLD2 wild‐type 3'‐UTR sequence containing the miR‐5132‐5p binding site was synthesized and cloned into the pGL‐3 promoter vector (PLD2‐WT). An additional reporting vector containing the corresponding mutation 3'‐UTR sequence, named PLD2‐MUT, was also constructed. These luciferase reporter vectors were cotransfected into AR42J cells with miR‐5132‐5p mimic or mimic‐NC. Forty‐eight hours after transfection, luciferase activity was analyzed using a dual‐luciferase reporter assay system (Promega).

### 2.7 Statistical analysis

Statistical analysis was performed using the SPSS Software (IBM). Data are expressed as mean ± standard deviation of at least three experiments. One‐way analysis of variance was performed, followed by Tukey's and Student's *t*‐test to analyze differences between groups. Differences were considered statistically significant at *p* < .05.

## 3 RESULTS

### 3.1 Cerulein treatment induced rat pancreatic AR42J cells inflammation and apoptosis

**Figure 1:**
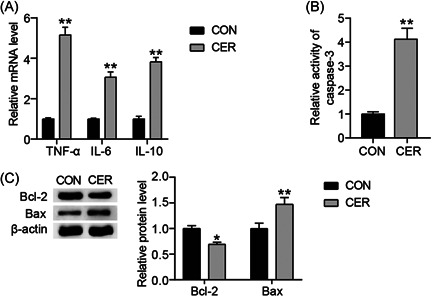
Cerulein treatment induced rat pancreatic AR42J cells inflammation and apoptosis. (A) RT‐qPCR test of three inflammatory mediators (TNF‐α, IL‐6, and IL‐10) after CER stimulation in AP cell model. (B) Caspase‐3 activity assay was performed to analyze caspase‐3 activity in AR42J cells treated with cerulein. (C) Wesern blot assay was performed to analyze Bax and Bcl‐2 protein levels in AR42J cells treated with cerulein. *p < .05; **p < .001 compared with CON group. CON, control; IL, interleukin; RT‐qPCR, reverse transcription‐quantitative polymerase chain; TNF, tumor necrosis factor.

To assess the mechanism of AP in vitro, we constructed a cerulein‐induced inflammation model of pancreatic acinar cells. Using RT‐qPCR, we detected levels of inflammatory cytokines, including tumor necrosis factor (TNF)‐α, interleukin (IL)−6, and IL‐10, in cell supernatants. Compared with the CON group, mRNA levels of TNF‐α, IL‐6, and IL‐10 in the CER group increased by 5.5‐, 3.5‐, and 4‐fold, respectively (Figure 1A). The CER group exhibited upregulated caspase‐3 activity (approximately 4‐fold) in AR42J cells when compared with that of the CON group (Figure 1B). In addition, levels of apoptosis‐related proteins, including Bax and Bcl‐2, were determined using Western blot analysis. Bax protein expression in the CER group was approximately 1.5‐fold higher than in the CON group, while Bcl‐2 protein reduction was approximately 70% lower than in the CON group (Figure 1C). Taken together, these data demonstrate cerulein‐induced pancreatic acinar cell inflammation and apoptosis, indicating the successful establishment of an in vitro model of cerulein‐induced AP.

### 3.2 PLD2 was downregulated in AP, and PLD2 overexpression reduced cerulein‐induced inflammation and apoptosis of AR42J cells via the Nrf2/NFκB pathway

**Figure 2:**
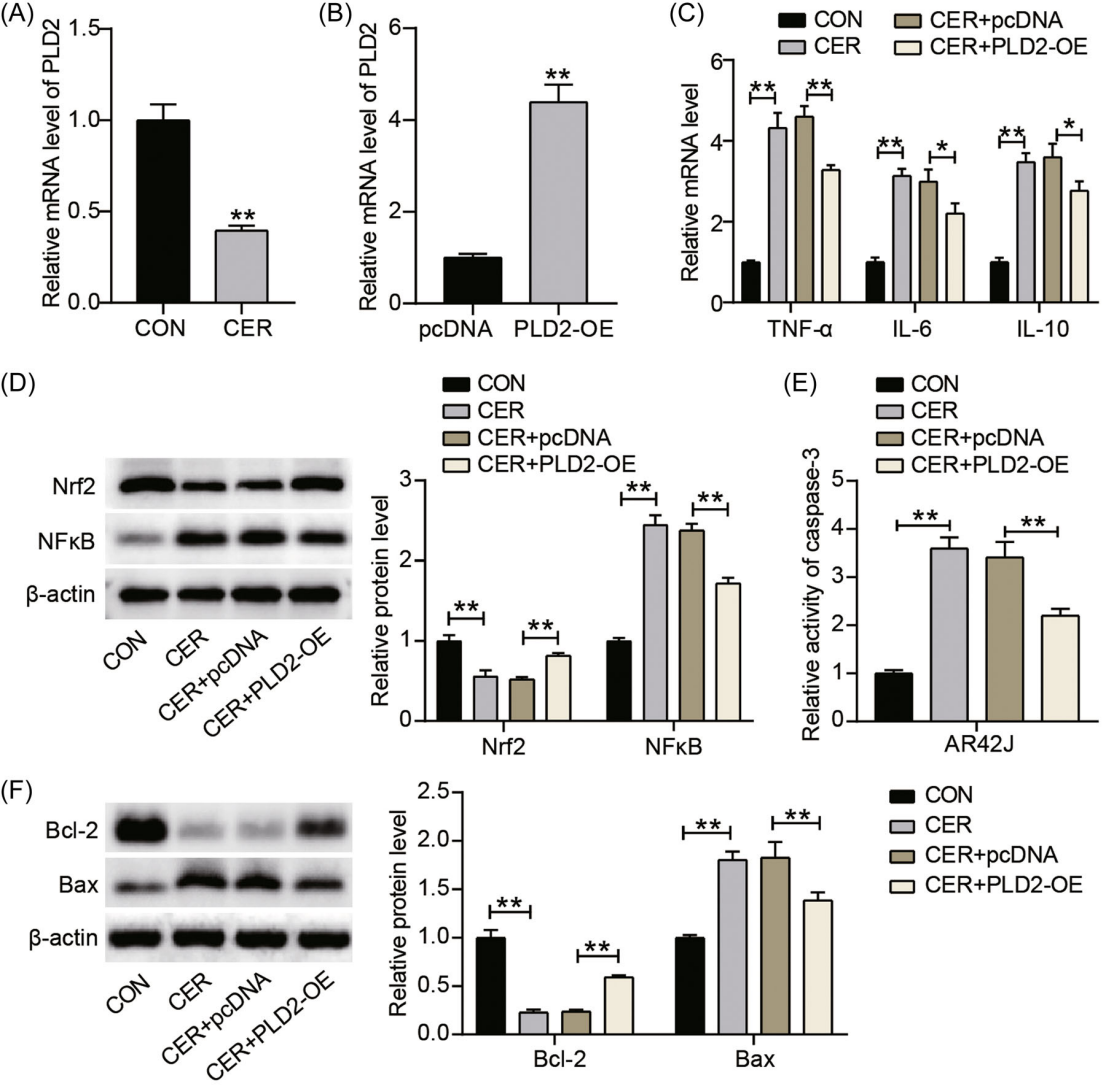
PLD2 was downregulated in AP, and its overexpression reduced inflammation and apoptosis of AR42J cells induced by cerulein via Nrf2/NFκB pathway. (A) RT‐qPCR test of PLD2 mRNA expression after CER stimulation in AR42J cells treated with cerulein. **p < .001 compared with CON group. (B) PLD2 mRNA expression in AR42J cells transfected with PLD2‐OE was measured by RT‐qPCR assay. **p < .001 compared with pcDNA group. (C). RT‐qPCR test of three inflammatory mediators (TNF‐α, IL‐6, and IL‐10) in AR42J cells treated with cerulein and transfected with PLD2‐OE. *p < .05; **p < .001. (D) The protein levels of Nrf2 and NFκB in AR42J cells treated with cerulein and transfected with PLD2‐OE was detected by western blot assay. (E) Caspase‐3 activity assay was performed to analyze caspase‐3 activity in AR42J cells treated with cerulein and transfected with PLD2‐OE. **p < .001. (F) Western blot assay was performed to analyze Bax and Bcl‐2 protein levels in AR42J cells treated with cerulein and transfected with PLD2‐OE. **p < .001. CON, control; IL, interleukin; mRNA, messenger RNA; PLD2, phospholipase D2; RT‐qPCR, reverse transcription‐quantitative polymerase chain; TNF, tumor necrosis factor.

To examine the potential effects of PLD2 on AP, we determined PLD2 expression in cerulein‐treated AR42J cells using RT‐qPCR. The expression level of PLD2 in the CER group decreased by approximately 60% when compared with that in the CON group, indicating that PLD2 may be involved in AP progression (Figure 2A). Based on these results, plasmids overexpressing PLD2 were transfected into AR42J cells to investigate the effects of PLD2 on inflammation and apoptosis in AR42J cells. As shown in Figure 2B, THE level of PLD2 in the PLD2‐OE group was approximately 4.5‐fold higher than that in the pcDNA group. RT‐qPCR analysis revealed that mRNA expression levels of TNF‐α, IL‐6, and IL‐10 in the CER + PLD2‐OE group were significantly lower than those in the CER + pcDNA group (Figure 2C), indicating that PLD2 overexpression inhibited AP inflammation. Western blot analysis showed that Nrf2 expression in the PLD2‐OE group was upregulated, whereas NF‐κB expression in the PLD2‐OE group was downregulated (Figure 2D). In addition, the activity of caspase‐3 in cerulein‐treated AR42J cells decreased by 40% on upregulating PLD2 expression (Figure 2E). Furthermore, western blot analysis revealed that Bcl‐2 levels in the CER + PLD2‐OE group were 1.5‐fold higher than in the CER + pcDNA group, while Bax levels decreased by 30% (Figure 2F). These data suggest that overexpression of PLD2 could attenuate the proinflammatory and proapoptotic effects of cerulein on AR42J cells via the Nrf2/NFκB pathway.

### 3.3 miR‐5132‐5p targeted PLD2

**Figure 3:**
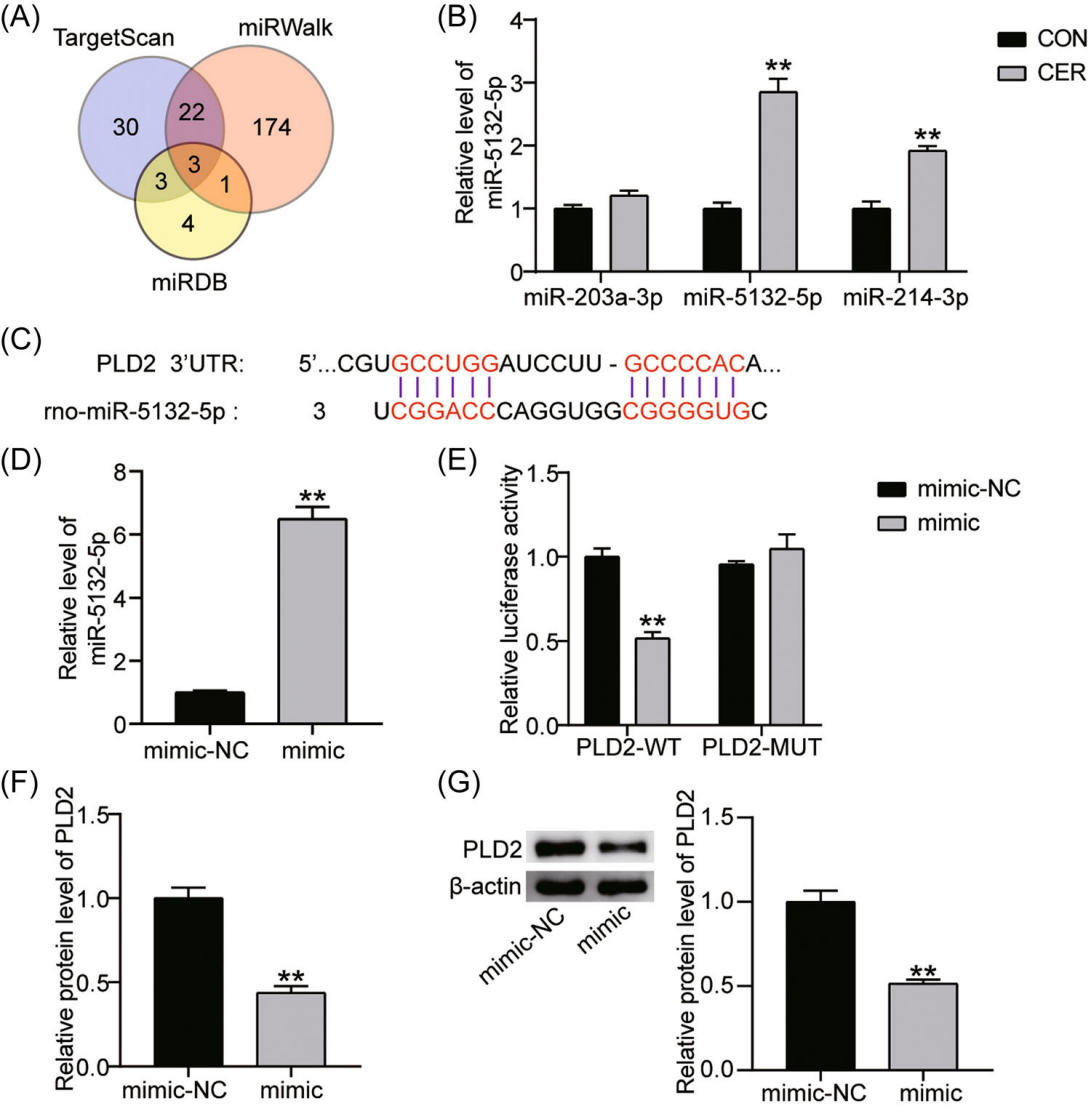
PLD2 targeted by miR‐5132‐5p. (A) Three miRNAs (miR‐203a‐3p, miR‐5132‐5p and miR‐214‐3p) were overlapped from TargetScan, miRWalk and miRDB. TargetScan, miRWalk and miRDB are the databases for predicting the upstream miRNAs of PLD2. (B) RT‐qPCR test of miR‐203a‐3p, miR‐5132‐5p and miR‐214‐3p expression in AR42J cells treated with cerulein. **p < .001 compared with CON group. (C) The complementary sequences between miR‐5132‐5p and PLD2 were predicted by TargetScan online database. (D) MiR‐5132‐5p expression in AR42J cells transfected with miR‐5132‐5p mimic was measured by RT‐qPCR assay. **p < .001 compared with mimic‐NC group. (E) The Luciferase activity of AR42J cells transfected with PLD2‐WT/PLD2‐MUT reporter and miR‐5132‐5p mimic/mimic‐NC was detected by dual luciferase reporter assay. **p < .001 compared with mimic‐NC group. (F) PLD2 mRNA expression in AR42J cells transfected with miR‐5132‐5p mimic was measured by RT‐qPCR assay. **p < .001 compared with mimic‐NC group. (G) PLD2 protein expression in AR42J cells transfected with miR‐5132‐5p mimic was measured by western blot assay. **p < .001 compared with mimic‐NC group. CON, control; IL, interleukin; mRNA, messenger RNA; PLD2, phospholipase D2; RT‐qPCR, reverse transcription‐quantitative polymerase chain; TNF, tumor necrosis factor.

Three online databases (TargetScan, miRWalk, and miRDB) were used to predict potential miRNAs capable of binding to PLD2. As shown in Figure 3A, miR‐203a‐3p, miR‐5132‐5p, and miR‐214‐3p were common miRNAs in all three databases. Therefore, the expression of the three miRNAs was detected after cerulein treatment. Based on RT‐qPCR results, expression levels of miR‐5132‐5p and miR‐214‐3p in the CER group increased 2.8‐ and 2‐fold when compared with those in the CON group, but the level of miR‐203a‐3p was not significantly altered (Figure 3B). Given the pronounced change in miR‐5132‐5p expression, this miRNA was selected for follow‐up studies. As shown in Figure 3C, miR‐5132‐5p and PLD2 displayed potential binding sites on the TargetScan website. In addition, miR‐5132‐5p expression increased by 6.3‐fold in AR42J cells transfected with the miR‐5132‐5p mimic (Figure 3D). Luciferase analysis revealed that the miR‐5132‐5p mimic cotransfected with PLD2‐WT reduced the luciferase activity in AR42J cells by approximately 50%, while the miR‐5132‐5p mimic cotransfected with PLD2‐MUT did not significantly impact luciferase activity (Figure 3E). This result confirmed the interaction between miR‐5132‐5p and the 3'‐UTR of PLD2. Additionally, we analyzed the miR‐5132‐5p mediated regulation of PLD2 expression. The results showed that miR‐5132‐5p mimic treatment reduced PLD2 mRNA (Figure 3F) and protein levels (Figure 3G) by 55% and 50%, respectively. Overall, miR‐5132‐5p could target PLD2 and negatively regulate its expression.

### 3.4 Upregulation of miR‐5132‐5p attenuated the alleviating effect of PLD2 overexpression on cerulein‐induced inflammation and apoptosis of AR42J cells

**Figure 4:**
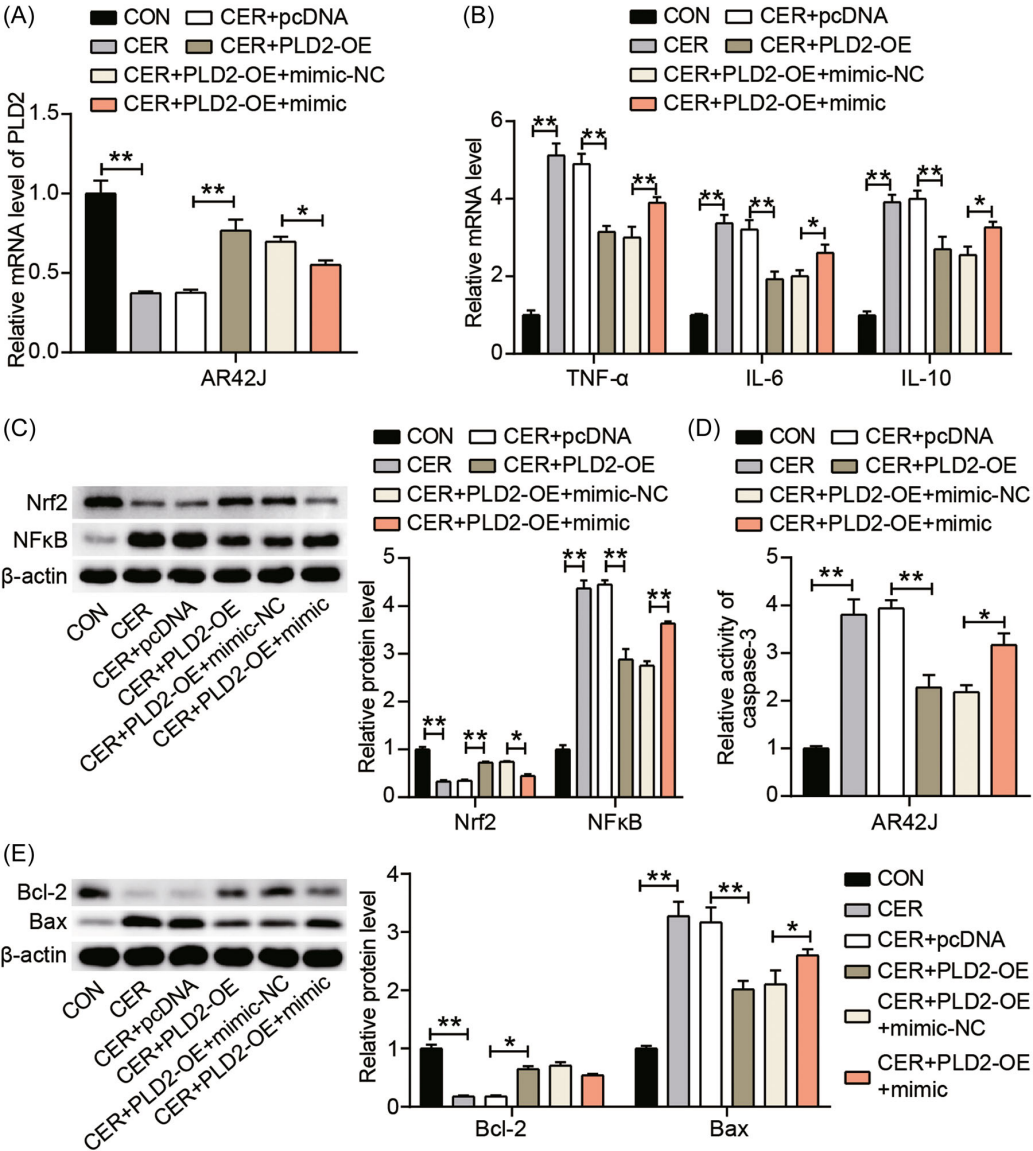
Upregulation of miR‐5132‐5p attenuated the alleviating effect of PLD2 overexpression on cerulein‐induced inflammation and apoptosis of AR42J cells via Nrf2/NFκB pathway. (A) PLD2 mRNA expression in AR42J cells transfected with CON, CER, CER + pcDNA, CER + PLD2‐OE, CER + PLD2‐OE + mimic‐NC or CER + PLD2‐OE + mimic was measured by RT‐qPCR assay. (B) RT‐qPCR test of three inflammatory mediators (TNF‐α, IL‐6, and IL‐10) in AR42J cells treated with cerulein and transfected with these above‐mentioned vectors. (C) Wesern blot assay was used to detect the protein levels of Nrf2 and NFκB in AR42J cells treated with cerulein and transfected with these above‐mentioned vectors. (D) Caspase‐3 activity assay was performed to analyze caspase‐3 activity in AR42J cells treated with cerulein and transfected with these above‐mentioned vectors. (E) Wesern blot assay was performed to analyze Bax and Bcl‐2 protein levels in AR42J cells treated with cerulein and transfected with these above‐mentioned vectors. *p < .05; **p < .001. CON, control; IL, interleukin; mRNA, messenger RNA; PLD2, phospholipase D2; RT‐qPCR, reverse transcription‐quantitative polymerase chain; TNF, tumor necrosis factor.

Based on the above findings, we demonstrated that miR‐5132‐5p could target PLD2 to inhibit PLD2 expression. We further investigated whether miR‐5132‐5p overexpression could regulate the effect of PLD2 overexpression on cerulein‐induced AP by transfecting miR‐5132‐5p mimic and PLD2‐OE into AR42J cells. Based on RT‐qPCR analysis, miR‐5132‐5p overexpression reversed the upregulated PLD2 expression in the PLD2‐OE group (Figure 4A). In addition, the analysis of TNF‐α, IL‐6, and IL‐10 showed that the miR‐5132‐5p mimic upregulated the reduced inflammation‐related factors induced by cerulein in AR42J cells transfected with PLD2‐OE (Figure 4B). The miR‐5132‐5p mimic reduced the enhanced Nrf2 expression induced by PLD2‐OE while increasing the PLD2‐OE‐induced decrease in NF‐κB expression (Figure 4C). Subsequently, compared with CER + PLD2‐OE + mimic‐NC, the activity of Caspase‐3 in the CER + PLD2‐OE + mimic group increased by approximately 1.3‐fold, indicating that the miR‐5132‐5p mimic partially eliminated the inhibitory effect of PLD2‐OE on apoptosis (Figure 4D). Furthermore, miR‐5132‐5p ectopic expression significantly enhanced Bax expression and reduced Bcl‐2 levels, and the inhibitory effect of PLD2‐OE on apoptotic proteins was eliminated (Figure 4E). Collectively, miR‐5132‐5p mimic reversed the inhibitory effects of PLD2 overexpression on cerulein‐induced inflammation and apoptosis in AR42J cells.

## 4 DISCUSSION

As a well‐known disease, AP may develop into a systemic disease involving a wide range of inflammatory responses and organ dysfunction, posing a substantial burden on the healthcare system.^17^, ^18^ Accumulated evidence suggests that the rapid release of proinflammatory and anti‐inflammatory mediators causes localized inflammatory responses in the tissues.^19^, ^20^ Previous studies have shown that TNF‐α is a key regulator of the AP inflammatory response by initiating acinar cell injury to induce an inflammatory response and mediate IL‐6 production and release.^21^ Moreover, IL‐10 levels represent the immunosuppressive phase.^22^ Cerulein has been shown to induce AP in vitro models.^23^ In the present study, we detected increased expression of TNF‐α, IL‐6, and IL‐10 in cerulein‐treated AR42J cells, accompanied by enhanced apoptosis. These findings are consistent with those of Sheng et al.^24^ demonstrating the successful establishment of an in vitro model of AP. The Nrf2/NFκB pathway has been associated with AP.^25^ Herein, we investigated the effects of PLD2 and miR‐5132‐5p on inflammatory levels and apoptosis in an AP model of cerulein‐induced AR42J cells via the regulation of the Nrf2/NFκB pathway. We observed that miR‐5132‐5p regulated PLD2 to promote apoptosis and inflammatory injury in AP in vitro.

Neutrophil infiltration is a key process of AP disease progression.^26^ Reportedly, PLD2 deficient sepsis mice exhibit notable characteristics, including improved survival, decreased organ damage, and increased neutrophil number in lung tissue.^27^ In addition, Wahida et al.^9^ have found that PLD2 knockout mice, an AP mouse model, show more neutrophils infiltrating the pancreatic tissue than wild‐type mice. In the present study, we found that PLD2 expression in cerulein‐treated AR42J cells was decreased. Additionally, PLD2 overexpression inhibited the inflammatory response and apoptosis of AR42J cells via the Nrf2/NFκB pathway. This is similar to the effect of PLD2 on intestinal inflammation^7^ and comprehensively reflects the therapeutic effect of PLD2 on AP.

MiRNAs participate in the regulation of AP via posttranscriptional repressor target genes. For example, miR‐325‐3p reduces inflammation, edema, bleeding, and necrosis in AP by targeting RIPK3.^28^ MiR‐20b‐5p regulates autophagy by directly targeting AKT3, inhibiting inflammation and apoptosis, and promoting angiogenesis, thereby attenuating severe AP.^29^ MiR‐339‐3p overexpression could regulate TRAF3 expression through the p38 pathway and inhibit cerulein‐induced AP cell inflammation and apoptosis.^30^ Furthermore, PLD2 was found to be a target gene of miR‐203^31^ and miR‐138.^32^ Therefore, we further examined miRNAs upstream of PLD2. MiR‐5132‐5p has rarely been reported, with only one study reporting that miR‐5132‐5p plays a crucial role in regulating drug‐induced liver injury and mediates hepatotoxicity.^33^ However, there is no direct evidence regarding its effect on inflammatory diseases or the pancreas. To the best of our knowledge, we, for the first time, revealed that miR‐5132‐5p could accelerate cerulein‐induced inflammation and apoptosis in AR42J cells. Targeting studies revealed that miR‐5132‐5p binds to the 3'‐UTR of PLD2 and plays a role in silencing PLD2 expression after transcription. These results suggest that miR‐5132‐5p could promote inflammation and apoptosis in cerulein‐treated AR42J cells by targeting PLD2.

One limitation of the present study is the lack of relevant results from animal models. In future studies, we plan to establish an in vivo AP model to verify the mechanism of action of miR‐5132‐5p/PLD2. In addition, the upstream regulatory factors of miR‐5132‐5p/PLD2 warrant further investigation.

## 5 CONCLUSION

Herein, we demonstrated that PLD2 was significantly downregulated and miR‐5132‐5p was significantly upregulated in cerulein‐treated AR42J cells. Further functional and mechanistic experiments showed that PLD2 was negatively regulated by miR‐5132‐5p, thus alleviating cerulein‐induced inflammatory injury and apoptosis of AR42J cells by regulating the Nrf2/NFκB pathway.

## AUTHOR CONTRIBUTIONS

Hailong Wu performed the experiments and data analysis. Hao Chen conceived and designed the study. Hao Chen did investigation. Rui Zhou wrote the paper. Hailong Wu reviewed and edited the manuscript. All authors read and approved the manuscript.

## CONFLICT OF INTEREST STATEMENT

The authors declare no conflict of interest.

## Supporting information

Supporting information.

Supporting information.

## Data Availability

All data generated or analyzed during this study are included in this article.

## References

[1] Boxhoorn L , Voermans RP , Bouwense SA , et al. Acute pancreatitis. Lancet. 2020;396(10252):726‐734. 32891214 10.1016/S0140-6736(20)31310-6

[2] Patel K , Trivedi RN , Durgampudi C , et al. Lipolysis of visceral adipocyte triglyceride by pancreatic lipases converts mild acute pancreatitis to severe pancreatitis independent of necrosis and inflammation. Am J Pathol. 2015;185(3):808‐819. 25579844 10.1016/j.ajpath.2014.11.019 PMC4348470

[3] Waller A , Long B , Koyfman A , Gottlieb M . Acute pancreatitis: updates for emergency clinicians. J Emerg Med. 2018;55(6):769‐779. 30268599 10.1016/j.jemermed.2018.08.009

[4] Wang GJ , Gao CF , Wei D , Wang C , Ding SQ . Acute pancreatitis: etiology and common pathogenesis. World J Gastroenterol. 2009;15(12):1427‐1430. 19322914 10.3748/wjg.15.1427 PMC2665136

[5] McDermott M , Wakelam MJ , Morris AJ . Phospholipase D. Biochem Cell Biol. 2004;82(1):225‐253. 15052340 10.1139/o03-079

[6] Ghim J , Chelakkot C , Bae YS , Suh PG , Ryu SH . Accumulating insights into the role of phospholipase D2 in human diseases. Adv Biolog Regul. 2016;61:42‐46. 10.1016/j.jbior.2015.11.010 26695710

[7] Zhou G , Yu L , Yang W , Wu W , Fang L , Liu Z . Blockade of PLD2 ameliorates intestinal mucosal inflammation of inflammatory bowel disease. Mediators Inflamm. 2016;2016:1‐14. 10.1155/2016/2543070 PMC5046040 27721573

[8] Abdulnour REE , Howrylak JA , Tavares AH , et al. Phospholipase D isoforms differentially regulate leukocyte responses to acute lung injury. J Leukoc Biol. 2018;103(5):919‐932. 29437245 10.1002/JLB.3A0617-252RR PMC6375305

[9] Ali WH , Chen Q , Delgiorno KE , et al. Deficiencies of the lipid‐signaling enzymes phospholipase D1 and D2 alter cytoskeletal organization, macrophage phagocytosis, and cytokine‐stimulated neutrophil recruitment. PLoS One. 2013;8(1):e55325. 23383154 10.1371/journal.pone.0055325 PMC3557251

[10] Xiang H , Tao X , Xia S , et al. Targeting MicroRNA function in acute pancreatitis. Front Physiol. 2017;8:726. 28983256 10.3389/fphys.2017.00726 PMC5613139

[11] Correia de Sousa M , Gjorgjieva M , Dolicka D , Sobolewski C , Foti M . Deciphering miRNAs' action through miRNA editing. Int J Mol Sci. 2019;20(24):6249. 31835747 10.3390/ijms20246249 PMC6941098

[12] Hu LH , Ji JT , Li ZS . Potential application of miRNAs as diagnostic and therapeutic tools in chronic pancreatitis. J Cell Mol Med. 2015;19(9):2049‐2057. 26149296 10.1111/jcmm.12603 PMC4568909

[13] Maltby S , Plank M , Tay HL , Collison A , Foster PS . Targeting MicroRNA function in respiratory diseases: mini‐review. Front Physiol. 2016;7:21. 26869937 10.3389/fphys.2016.00021 PMC4740489

[14] Tan Y , Zhang W , Wu H , et al. Effects of emodin on intestinal mucosal barrier by the upregulation of miR‐218a‐5p expression in rats with acute necrotizing pancreatitis. Int J Immunopathol Pharmacol. 2020;34:205873842094176. 10.1177/2058738420941765 PMC7364802 32664763

[15] Liu S , Zou H , Wang Y , et al. miR‐155‐5p is negatively associated with acute pancreatitis and inversely regulates pancreatic acinar cell progression by targeting rela and Traf3. Cell Physiol Biochem. 2018;51(4):1584‐1599. 30497068 10.1159/000495648

[16] Duan PY , Ma Y , Li XN , et al. Inhibition of RIPK1‐dependent regulated acinar cell necrosis provides protection against acute pancreatitis via the RIPK1/NF‐κB/AQP8 pathway. Exp Mol Med. 2019;51(8):1‐17. 10.1038/s12276-019-0278-3 PMC6802613 31375658

[17] Peery AF , Crockett SD , Murphy CC , et al. Burden and cost of gastrointestinal, liver, and pancreatic diseases in the United States: update 2018. Gastroenterology. 2019;156(1):254‐272. 30315778 10.1053/j.gastro.2018.08.063 PMC6689327

[18] Yadav D , Lowenfels AB . The epidemiology of pancreatitis and pancreatic cancer. Gastroenterology. 2013;144(6):1252‐1261. 23622135 10.1053/j.gastro.2013.01.068 PMC3662544

[19] Lankisch PG , Apte M , Banks PA . Acute pancreatitis. Lancet. 2015;386(9988):85‐96. 25616312 10.1016/S0140-6736(14)60649-8

[20] Singh P , Garg PK . Pathophysiological mechanisms in acute pancreatitis: current understanding. Indian J Gastroenterol. 2016;35(3):153‐166. 27206712 10.1007/s12664-016-0647-y

[21] Meher S , Mishra TS , Sasmal PK , et al. Role of biomarkers in diagnosis and prognostic evaluation of acute pancreatitis. J Biomark. 2015;2015:1‐13. 10.1155/2015/519534 PMC4541003 26345247

[22] Dambrauskas Z , et al. Different profiles of cytokine expression during mild and severe acute pancreatitis. World J Gastroenterol. 2010;16(15):1845‐1853. 20397261 10.3748/wjg.v16.i15.1845 PMC2856824

[23] Sledzinski M , Borkowska A , Sielicka‐Dudzin A , et al. Cerulein‐induced acute pancreatitis is associated with c‐Jun NH(2)‐terminal kinase 1‐dependent ferritin degradation and iron‐dependent free radicals formation. Pancreas. 2013;42(7):1070‐1077. 23921964 10.1097/MPA.0b013e318287d097

[24] Sheng B , Zhao L , Zang X , et al. Quercetin inhibits caerulein‐induced acute pancreatitis through regulating miR‐216b by targeting MAP2K6 and NEAT1. Inflammopharmacology. 2021;29(2):549‐559. 33051781 10.1007/s10787-020-00767-7

[25] Pasari LP , Khurana A , Anchi P , Aslam Saifi M , Annaldas S , Godugu C . Visnagin attenuates acute pancreatitis via Nrf2/NFκB pathway and abrogates associated multiple organ dysfunction. Biomed Pharmaco. 2019;112:108629. 10.1016/j.biopha.2019.108629 30798137

[26] Merza M , Hartman H , Rahman M , et al. Neutrophil extracellular traps induce trypsin activation, inflammation, and tissue damage in mice with severe acute pancreatitis. Gastroenterology. 2015;149(7):1920‐1931. 26302488 10.1053/j.gastro.2015.08.026

[27] Lee SK , Kim SD , Kook M , et al. Phospholipase D2 drives mortality in sepsis by inhibiting neutrophil extracellular trap formation and down‐regulating CXCR2. J Exp Med. 2015;212(9):1381‐1390. 26282875 10.1084/jem.20141813 PMC4548059

[28] Jia A , Yang ZW , Shi JY , Liu JM , Zhang K , Cui YF . MiR‐325‐3p alleviates acute pancreatitis via targeting RIPK3. Dig Dis Sci. 2022;67(9):4471‐4483. 35094251 10.1007/s10620-021-07322-6

[29] Tang GX , Yang MS , Xiang KM , Yang BC , Liu ZL , Zhao SP . MiR‐20b‐5p modulates inflammation, apoptosis and angiogenesis in severe acute pancreatitis through autophagy by targeting AKT3. Autoimmunity. 2021;54(7):460‐470. 34402705 10.1080/08916934.2021.1953484

[30] Wang Q , Liu S , Han Z . miR‐339‐3p regulated acute pancreatitis induced by caerulein through targeting TNF receptor‐associated factor 3 in AR42J cells. Open Life Sci. 2020;15(1):912‐922. 33817278 10.1515/biol-2020-0084 PMC7874543

[31] Chen Z , Li D , Cheng Q , et al. MicroRNA‐203 inhibits the proliferation and invasion of U251 glioblastoma cells by directly targeting PLD2. Mol Med Rep. 2014;9(2):503‐508. 24270883 10.3892/mmr.2013.1814

[32] Li HQ , Ye JH , Ding X , Wu HM . [Effect of mi‐138 targeting PLD2 gene on proliferation and migration of oral cancer cells]. Shanghai Kou Qiang Yi Xue. 2020;29(1):25‐30. 32524116

[33] Yang R , Bai Q , Zhang J , Sheng Y , Ji L . The altered liver microRNA profile in hepatotoxicity induced by rhizome *Dioscorea bulbifera* in mice. Hum Exp Toxicol. 2017;36(8):823‐832. 27609015 10.1177/0960327116666651

