# Supplementary material for: Phospholipase D2 targeted by miR‐5132‐5p alleviates cerulein‐induced acute pancreatitis via the Nrf2/NFκB pathway
Source: Immun Inflamm Dis. 2023 May 29;11(5):e831. doi: 10.1002/iid3.831 (PMC10227361; doi:10.1002/iid3.831)
Supplement: Supplementary file 1 — Supporting information. [file IID3-11-e831-s001.pdf]

editage

Editing Certificate

This document certifies that the manuscript listed below has been edited to ensure language and grammar accuracy and is error free in these aspects. The logical presentation of ideas and the structure of the paper were also checked during the editing process. The edit was performed by professional editors at Editage, a division of Cactus Communications. The author's core research ideas were not altered in any way during the editing process. The quality of the edit has been guaranteed, with the assumption that our suggested changes have been accepted and the text has not been further altered without the knowledge of our editors.

MANUSCRIPT TITLE

Phospholipase D2 targeted by miR-5132-5p alleviates cerulein-induced acute pancreatitis via the Nrf2/NFκB pathway

AUTHORS

Hailong Wu

ISSUED ON

January 11, 2023

JOB CODE

RQIUR_154

THE EDITAGE Promise QUALITY GUARANTEE

Vikas Narang

Vikas Narang
Chief Operating Officer - Editage

editage

Editage, a brand of Cactus Communications, offers professional English language editing and publication support services to authors engaged in over 1300 areas of research. Through its community of experienced editors, which includes doctors, engineers, published scientists, and researchers with peer review experience, Editage has successfully helped authors get published in internationally reputed journals. Authors who work with Editage are guaranteed excellent language quality and timely delivery.

GLOBAL :
+1(833) 979-0061 | request@editage.com

CHINA :
400-120-3020或021-6020-9400 | fabiao@editage.cn

CACTUS

IMPACT SCIENCE impact.science

researcher.life

CACTUS LIFE SCIENCES lifesciences.cactusglobal.com
